# A Novel Variant of Type II Monteggia Equivalent Fracture-Dislocation in Children: A Case Report

**DOI:** 10.7759/cureus.19541

**Published:** 2021-11-13

**Authors:** Ali R Karashi, Sadaf M Basheer, Faris M Alsobyani, Mohamed I Janahi, Amna M Albu Mahmud, Abdulrahman I Janahi

**Affiliations:** 1 Pediatric Orthopedics, Salmaniya Medical Complex, Manama, BHR; 2 Internal Medicine, Salmaniya Medical Complex, Manama, BHR; 3 Orthopedic Surgery, Salmaniya Medical Complex, Manama, BHR; 4 Medicine, Salmaniya Medical Complex, Manama, BHR; 5 Department of Surgery, Arabian Gulf University, Manama, BHR

**Keywords:** monteggia lesions, fracture fixation, monteggia equivalents, physeal injury, elbow injury

## Abstract

Monteggia fracture-dislocations are extremely rare in children. By definition, it is an ulnar shaft fracture with proximal radioulnar joint dislocation. Throughout the years, various equivalents of this entity have been described. In this report, we present a unique case of a type II Monteggia fracture equivalent with an ipsilateral fracture of the proximal radius and olecranon in a child. The patient was a 12-year-old male who presented with a history of a fall on an outstretched hand. The diagnosis was made based on the clinical examination and plain radiographs. We describe the management of this unique fracture and discuss the possible mechanism of injury. We have highlighted a rare combination of injuries. It is crucial to investigate the condition properly in order to avoid missing the diagnosis and to prevent poor outcomes and further unnecessary revision surgeries.

## Introduction

Monteggia fracture-dislocations by definition involve a fracture of shaft of ulna along with dislocation of proximal radioulnar joint [1]. While distal forearm fractures can be grouped as the most common fracture in the pediatric population, Monteggia lesions are rare, accounting for 0.4% of all fractures in this age group. Initially described by Giovanni Battista, it was Bado who provided more insight into the lesion, by classifying the injury into classical Monteggia and Monteggia equivalent or variants [1].

Throughout the years, our improved understanding of the elbow and forearm trauma has led us to distinguish classical ulna fracture-dislocations from other injuries that are often grouped under the terminology of Monteggia equivalents [2,3]. The injuries classified under this category are not clearly understood and the studies in the literature are still divided in terms of a clear definition of what a Monteggia equivalent lesion would constitute. However, it has been observed that as such the lesions that involve the radial head in addition to the ulna fracture are associated with worse outcomes as compared to classical Monteggia lesions [4], thus making it imperative for us to recognize this subset of lesions with increasing vigilance to provide optimal outcomes.

We present a rare fracture pattern combination of what could be described as an additional variant of type II Monteggia equivalent that involves olecranon fracture with type I Salter-Harris radial neck fracture and posterior dislocation of the elbow joint. To the best of our knowledge, such a fracture combination has not been previously described in the literature.

## Case presentation

**Figure 1:**
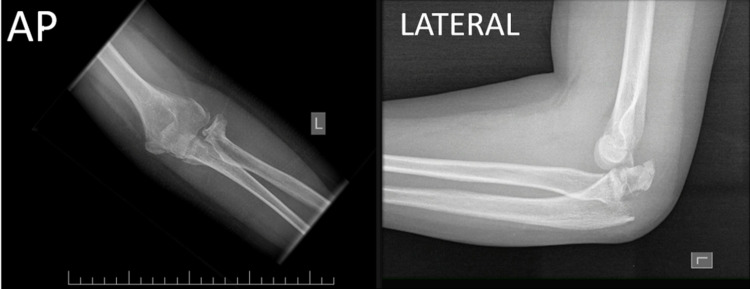
Displaced intraarticular fracture of olecranon with posterior dislocation of the left elbow and displaced fracture of radial neck SH type 1 SH: Salter-Harris; AP: anteroposterior

Our patient was a 12-year-old Bahraini male who was seen initially in the A&E with a history of a fall from a height of around 2 meters on an outstretched hand. He had an obvious deformity of his elbow joint with an intact soft tissue envelope and no distal neurovascular deficits. Plain radiographs revealed a closed fracture-dislocation involving the olecranon process, a Salter-Harris type I fracture of the radial neck, and posterior dislocation of the elbow joint (Figure 1).

**Figure 2:**
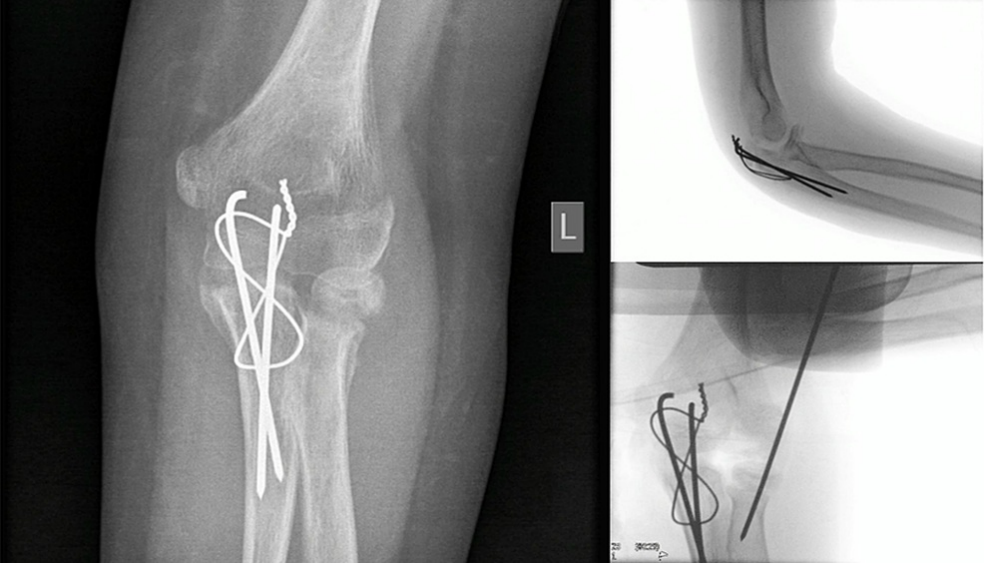
Tension band construct used for olecranon fixation with K-wire fixation of the radial neck fracture

Open reduction and internal fixation of the fracture were undertaken the following day, under general anesthesia and in the lateral position; the elbow was approached posteriorly. The radial head was indirectly reduced and held using a 1.6-mm K-wire; the olecranon was reduced under direct vision and held with a tension band using two 1.6-mm K-wires (Figure 2).

**Figure 3:**
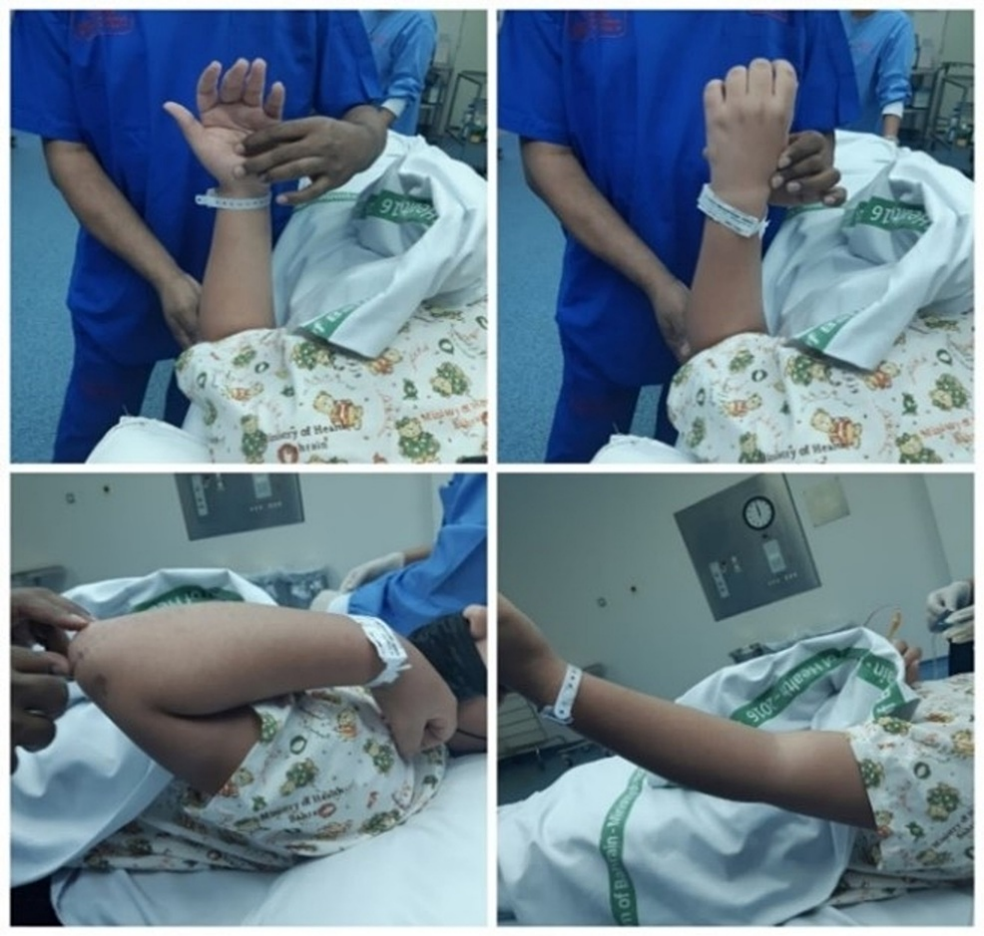
Normal range of motion after four months of surgery

Postoperatively, the elbow was protected using an above-elbow slab for three weeks, after which both active and passive elbow range of motion (ROM) was initiated. The fracture healed uneventfully, and the patient had a complete ROM of 30-130 degrees at the elbow joint within six weeks of fixation. The implant was removed after six months. The patient was completely asymptomatic with a full range of flexion, extension, supination, and pronation along with the evidence of bony union (Figure 3).

## Discussion

Jose Luis Bado coined the term Monteggia-like lesion in 1957 to describe the complex trauma to the elbow joint involving proximal ulna fracture and radial head dislocation [1]; however, additional varieties such as radiohumeral, ulnohumeral, and distal radioulnar dislocations have also been described [2,5,6].

There is a paucity of data in the literature to determine which injuries are to be included in Monteggia variant lesions. Bado has given a numerical classification of classic Monteggia lesions and grouped a number of fracture patterns under class I, which he described as Monteggia equivalents based on a similar mechanism of injury [1] and their similar management though it has been universally accepted that this subset of injuries is associated with poor outcomes as compared to conventional Monteggia fracture-dislocation [4,7] (Table 1).

**Table 1:** Various Monteggia lesions and proposed mechanism of injury

Direction of radial head dislocation	Ulna fracture	Mechanism of injury	Incidence
Type I anterior	Anterior angulation, usually midshaft	Hyperextension, hyperpronation, direct blow	~70%
Type II posterior	Posterior angulation, diaphyseal or metaphyseal	Hyperflexion	~3%–5%
Type III lateral or anterolateral	Lateral angulation, metaphyseal, usually greenstick	Hyperextension, lateral varus stress	~23%–26%
Type IV anterior, with fracture radius shaft	Diaphyseal	Hyperpronation	<1%

The mechanism of injury proposed regarding Monteggia lesions is hyperpronation [8] injury; however, there are multiple theories that describe a combination of forces causing an intrinsically unstable fracture of ulnar diaphysis along with ligamentous disruption of the radial head causing a radial head dislocation [1,2,8].

Since its description, various variants have been studied, of which the posterior variant type is the most common [4,5,7,9,10]. The original account of posterior variant type includes ulnar diaphysis fracture along with a fracture of the radial head that occurs as the radial head collides with capitellum as it dislocates posteriorly [7] (Table 2).

**Table 2:** Various fracture patterns under Monteggia equivalents

Pattern	Description
Type I	Isolated anterior dislocation of the radial head (with plastic deformation of ulna). Isolated radial neck fracture. Pulled elbow syndrome. Fracture of the ulnar diaphysis with fracture of the radial neck. Fractures of both bones in the forearm (wherein the radial fracture is above the junction of the proximal and the middle third). Fracture of ulnar diaphysis with anterior dislocation of the radial head and an olecranon fracture. Fracture of the ulnar diaphysis (at the proximal and middle third junction) with displaced extension type supracondylar fracture of the humerus. Fracture of olecranon with Salter-Harris type I physeal injury to the proximal radius
Type II	Monteggia equivalent: posterior elbow dislocation in children
Type III	Monteggia equivalent: oblique fracture of the ulna (with varus malalignment) with displaced fracture of the lateral condyle of the humerus
Type IV	Monteggia equivalent: distal humerus fracture with proximal third ulnar diaphysis fracture and distal radial metaphyseal fracture with anterior dislocation of the radial head

Our case represents a further extension of this variety of Monteggia variants where instead of ulnar diaphysis fracture there is a proximal ulnar metaphysis (olecranon) fracture along with Salter-Harris type I radial neck fracture and posterior dislocation of the elbow joint; the proximal radioulnar joint in this situation remains intact. It occurs due to falling on an outstretched hand with the hand going into forced pronation. This subtype holds importance due to its rarity and hence the ease with which it can be missed. The literature reports similar anterior and lateral lesions with radial head and neck fracture.

The Monteggia variant lesion poses a challenge even to the experienced surgeon, and the final outcome of the injury is dictated by the comminution and direction of dislocation. An accurate diagnosis entailing the exact extent of the injury is essential for optimal management. A complete ROM at the elbow joint and stability can only be achieved with meticulous operative therapy aiming at anatomical reduction of the joint [9,11].

Bado type I injuries are universally associated with good functional outcomes due to the low incidence of concomitant injuries, but Bado type II, the one associated with a posterior dislocation, have been reported to show significantly poorer outcomes, which makes it even more important to accurately recognize the magnitude of the injury [3,5,10].

## Conclusions

Monteggia variants can be described as complex elbow injuries. Accurate preoperative diagnosis in terms of assessing the fracture and associated ligamentous disruption is crucial. One should aim for stable, anatomically reduced joint to achieve normal ROM and allow for early rehabilitation.

Through our case report, we aimed to highlight a rare combination of injuries, which, if appropriately recognized and managed, can be resolved appropriately, thereby preventing poor functional outcomes and high rates of revision surgeries.
